# Efficacy, safety, and tolerability of adjunctive perampanel in patients from China with focal seizures or generalized tonic‐clonic seizures: Post hoc analysis of phase III double‐blind and open‐label extension studies

**DOI:** 10.1111/cns.13458

**Published:** 2020-09-08

**Authors:** Liao Weiping, Zhou Dong, Hong Zhen, Anna Patten, Amitabh Dash, Manoj Malhotra

**Affiliations:** ^1^ Institute of Neurosciences and Department of Neurology of the Second Affiliated Hospital of Guangzhou Medical University Guangdong China; ^2^ West China Hospital Sichuan China; ^3^ Shanghai Huashan Hospital (affiliated to Fudan University) Shanghai China; ^4^ Eisai Ltd. Hatfield UK; ^5^ Eisai Singapore Pte. Ltd. Singapore; ^6^ Eisai Inc. Woodcliff Lake NJ USA

**Keywords:** Chinese, focal seizures, generalized tonic‐clonic seizures, perampanel, seizure freedom

## Abstract

**Aims:**

This post hoc analysis assessed the efficacy and safety/tolerability of adjunctive perampanel in patients from China (aged ≥12 years) with focal seizures (FS), with/without focal to bilateral tonic‐clonic seizures (FBTCS), or generalized tonic‐clonic seizures (GTCS).

**Methods:**

Study centers in China were identified using data from five double‐blind, randomized, phase III studies of adjunctive perampanel (2‐12 mg/day) and their open‐label extensions (OLEx). Efficacy assessments included median percent reduction in seizure frequency per 28 days, and 50% and 75% responder and seizure‐freedom rates. Safety/tolerability assessments included monitoring of treatment‐emergent adverse events (TEAEs).

**Results:**

Overall, 277 patients (placebo, n = 79; perampanel, n = 198) were included in the double‐blind safety analysis set. The full analysis set comprised 274 patients (FS, n = 238 [placebo, n = 60; perampanel, n = 178]; FBTCS, n = 120 [placebo, n = 31; perampanel, n = 89]; GTCS, n = 36 [placebo, n = 18; perampanel, n = 18]). Median percent reductions in seizure frequency for placebo vs perampanel were as follows: 16.6% vs 32.4% (FS; *P* < 0.05) and 39.1% vs 48.2% (FBTCS; not significant [NS]) at 4‐12 mg/day, and 37.9% vs 82.6% (GTCS; NS) at 8 mg/day; 50% responder rates were 31.7% vs 37.4% (FS; NS), 48.4% vs 51.9% (FBTCS; NS), and 33.3% vs 61.1% (GTCS; NS), respectively. Seizure‐freedom rates were 1.7% vs 9.2%, 16.1% vs 25.3%, and 16.7% vs 44.4%, respectively (all NS). Overall, 262 patients entered the OLEx (FS, n = 228; GTCS, n = 34). Perampanel was efficacious for up to four years for FS and FBTCS and up to two years for GTCS. Across the double‐blind and OLEx studies, TEAEs were reported in 65.7% and 81.3% of perampanel‐treated patients, respectively; the most common was dizziness. Efficacy and safety/tolerability outcomes were generally similar between Chinese and non‐Chinese patients.

**Conclusion:**

Adjunctive perampanel (up to 12 mg/day) may be a suitable treatment for Chinese patients with FS, with/without FBTCS, or GTCS, with similar efficacy and safety/tolerability compared to non‐Chinese patients.

## Graphical abstract

- Adjunctive perampanel was associated with greater median percent reductions in seizure frequency per 28 days vs placebo in Chinese patients with focal or generalized seizure types during double‐blind, randomized, phase III studies.
- During OLEx studies, adjunctive perampanel showed efficacy for up to 4 years for FS and FBTCS, and up to 2 years for GTCS.
- Perampanel was generally well tolerated during short‐ and long‐term treatment.

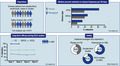

## 1 INTRODUCTION

Epilepsy is a chronic brain disease affecting approximately 50 million people worldwide.^1^ Asia includes >40 countries that are heterogeneous in geography, population, socioeconomic development, and healthcare systems.^2^ It is estimated that 23 million people living in Asia have epilepsy.^2^ However, epilepsies are associated with high economic, social, and psychological burdens in Asia given that stigma and discrimination against people with epilepsy are common.^1^, ^3^ The prevalence and incidence of epilepsy vary between countries. In China, prevalence and incidence rates are estimated as 4.6‐7.0 in 1000 and 28.8‐35.0 in 100 000, respectively, whereas in India these are 3.0‐11.9 in 1000 and 38.0‐60.0 in 100 000, respectively.^2^, ^4^, ^5^

There is a lack of epilepsy research in Asia, and efficacy and safety of new antiseizure medications (ASMs), which are the primary treatment option for patients with epilepsy, are often studied in patients from Europe and the United States.^3^ However, results of clinical trials in European or North American populations do not always translate to Asian populations.^3^, ^6^ It is important to consider ethnicity when evaluating ASMs, as patients of different ethnic or racial backgrounds may experience differences in their responses to ASMs due to intrinsic, extrinsic, or genetic factors, which could affect dosing recommendations, efficacy, and safety.^6^ Access to ASMs varies across Asia and newer ASMs with more favorable safety profiles may not be available in all countries.^3^ Furthermore, licensing of new ASMs in Asia is completed by individual countries and not by a continent‐wide regulatory body.^2^ When evaluating the efficacy and safety of ASMs, it is important to provide data at the individual country level where possible.

Perampanel, an orally active, noncompetitive, selective α‐amino‐3‐hydroxy‐5‐methyl‐4‐isoxazolepropionic acid (AMPA) receptor antagonist,^7^, ^8^ is approved as adjunctive therapy for focal seizures (FS, previously partial‐onset), with or without focal to bilateral tonic‐clonic seizures (FBTCS; previously secondarily generalized), in patients aged ≥12 years in >55 countries, and generalized tonic‐clonic seizures (GTCS; previously primary generalized tonic‐clonic) in patients aged ≥12 years in >50 countries (data on file, Eisai Inc., Woodcliff Lake, NJ, USA). Adjunctive perampanel is approved in several Asian countries for FS, with or without FBTCS, and/or GTCS (including India [data on file, Eisai Inc., Woodcliff Lake, NJ, USA], Indonesia,^9^ Japan [data on file, Eisai Co., Ltd., Tokyo, Japan], Malaysia,^10^ Philippines,^11^ Singapore,^12^ Taiwan,^13^ and Thailand^14^). Following a recent New Drug Application,^15^, ^16^ perampanel was also recently approved for use in China as an adjunctive treatment of FS, with or without FBTCS, in patients aged ≥12 years.^17^

The clinical development of adjunctive perampanel included phase III, double‐blind, randomized, placebo‐controlled studies in patients aged ≥12 years with FS (with or without FBTCS; international Studies 304 [NCT00699972], 305 [NCT00699582], and 306 [NCT00700310], and Asia‐Pacific Study 335 [NCT01618695]^18^, ^19^, ^20^, ^21^) or idiopathic generalized epilepsy and GTCS (international Study 332 [NCT01393743]^22^). Since randomized trials offer relatively short exposures to investigational ASMs (~8‐12 weeks), longer‐term follow‐up and postmarketing studies are important to assess long‐term efficacy and adverse side effects that may only occur after long‐term exposure.^23^ The long‐term efficacy and safety of adjunctive perampanel were assessed in patients who completed the phase III studies during open‐label extension (OLEx) studies: OLEx Study 307 (NCT00735397),^24^ Study 335 OLEx (NCT01618695; data on file, Eisai Co., Ltd.), and Study 332 OLEx (NCT01393743; data on file, Eisai Inc., Woodcliff Lake, NJ, USA).

To assess the efficacy, safety, and tolerability of adjunctive perampanel in patients from China, we performed a post hoc analysis in patients with FS, with or without FBTCS, or GTCS who participated in the double‐blind and OLEx studies at centers in China, compared with patients at the remaining centers (non‐Chinese). These analyses will provide guidance on long‐term perampanel use in China and support the recent approval of adjunctive perampanel for FS. They will also provide data for the use of adjunctive perampanel for GTCS in China.

## 2 METHODS

### 2.1 Study designs

For this post hoc analysis, Studies 306, 335, and 332 were identified as having centers in China. The designs of these studies have been previously reported^20^, ^21^, ^22^ and an overview is provided in Table S1. OLEx Study 307, Study 335 OLEx, and Study 332 OLEx also included centers in China (Table S1).

All studies were performed in accordance with the relevant Good Clinical Practice Guidelines. Trial protocols, amendments, and informed consent were reviewed by national regulatory authorities and independent ethics committees or institutional review boards. All patients gave written informed consent before participation.^20^, ^21^, ^22^, ^24^

### 2.2 Post hoc efficacy assessments

Efficacy assessments were based on the full analysis set and split by seizure type (FS, FBTCS, and GTCS). For the double‐blind study analyses, the full analysis set comprised all patients who received ≥1 dose of study drug (placebo or perampanel) and had any seizure frequency data during the double‐blind treatment phase. For the OLEx study analyses, the full analysis set comprised all patients who received ≥1 dose of perampanel during the OLEx study, and had baseline seizure frequency data, and any valid seizure data during perampanel treatment (defined below).

Efficacy assessments for up to four years (FS and FBTCS) or up to two years (GTCS) included the following: median percent change in seizure frequency per 28 days relative to double‐blind or preperampanel baseline (defined below); 50% and 75% responder rates (defined as the proportion of patients with a ≥50% or ≥75% reduction in seizure frequency per 28 days during the double‐blind study maintenance period or during each respective year of the perampanel treatment duration; last observation carried forward [LOCF]); and seizure‐freedom rates. For the double‐blind study analyses, seizure freedom was defined as the proportion of patients who were study completers and had no seizures during the double‐blind maintenance period; for the OLEx study analyses, this was the proportion of patients who completed the period of analysis and were free from seizures during that period of the perampanel treatment duration.

During the double‐blind studies, all efficacy assessments were performed for placebo vs perampanel 2, 4, 8, and 12 and 4‐12 mg/day combined for focal and FBTCS, and for placebo vs perampanel 8 mg/day for GTCS. During the OLEx studies, all patients received perampanel; therefore, no placebo comparison was included.

### 2.3 Post hoc safety and tolerability assessments

Safety and tolerability assessments were based on the safety analysis set. For the double‐blind study analyses, the safety analysis set comprised all patients who received ≥1 dose of study drug and had ≥1 postdose safety assessment. For the OLEx study analyses, the safety analysis set comprised all patients who received ≥1 dose of perampanel during the OLEx study and had any on‐treatment safety data during the OLEx study.

For assessment of safety and tolerability, data were pooled for all seizure types. Analyses included monitoring of treatment‐emergent adverse events (TEAEs), serious TEAEs, and TEAEs leading to discontinuation. A TEAE was defined as an adverse event with an onset date, or worsening in severity from baseline (pretreatment), on or after the first dose of study drug up to 30 days following study drug discontinuation.

### 2.4 Statistical analyses

The perampanel treatment duration started from the first dose of perampanel in the double‐blind study to the last dose of perampanel in the OLEx period, except for patients who had a gap in perampanel exposure from the double‐blind study to the OLEx period of >14 days; for these patients, the perampanel treatment duration was the OLEx exposure.

For patients who received placebo during the double‐blind studies, preperampanel baseline included seizure diary data collected during the double‐blind study. For patients who received perampanel during the double‐blind studies, preperampanel baseline included seizure diary data collected during the baseline period (prerandomization monitoring phase) of the double‐blind study plus 4 weeks prior.

For the double‐blind study analysis, median difference to placebo and 95% confidence intervals (CIs) was based on the Hodges‐Lehmann method. *P*‐values for median percent change were based on a rank analysis of covariance with treatment as a factor and prerandomization seizure frequency as a covariate, and for responder/seizure‐freedom rates were based on nonmissing values from a Cochran‐Mantel‐Haenszel test.

For OLEx analyses and to account for patients who dropped out of the study early, sensitivity analyses were conducted for efficacy assessments. For these, the LOCF approach was used, meaning that patients who completed or withdrew from the study had their last year of treatment carried forward to later time points; for patients who were treated for <1 year, their entire treatment period was carried forward to later time points.

## 3 RESULTS

### 3.1 Double‐blind studies: patients

Across double‐blind studies, 277 patients were identified from centers in China and included in the pooled safety analysis set (placebo, n = 79; perampanel, n = 198). Patient demographics and baseline characteristics were generally similar between the placebo and perampanel groups (Table 1). The most common seizure type during baseline in the placebo and perampanel groups was focal impaired awareness seizures (FIAS; previously complex partial). Most patients were receiving two or three concomitant ASMs during baseline (placebo, n = 35 [44.3%] and n = 22 [27.8%]; perampanel, n = 96 [48.5%] and n = 54 [27.3%], respectively); 48 (60.8%) placebo‐treated and 112 (56.6%) perampanel‐treated patients were receiving an enzyme‐inducing ASM (EIASM; carbamazepine, oxcarbazepine, phenytoin, and eslicarbazepine). The most common non‐EIASMs during baseline were valproic acid (placebo, n = 38 [48.1%]; perampanel, n = 103 [52.0%]), lamotrigine (placebo, n = 18 [22.8%]; perampanel, n = 60 [30.3%]), and levetiracetam (placebo, n = 20 [25.3%]; perampanel, n = 40 [20.2%]); the most common EIASM was carbamazepine (placebo, n = 30 [38.0%]; perampanel, n = 69 [34.8%]).

**TABLE 1:** Chinese patient demographics and baseline characteristics during the double‐blind and OLEx studies (safety analysis set)

	Double‐blind studies						OLEx studies
	Placebo (n = 79)	Perampanel					Perampanel (n = 262)
		2 mg/day (n = 15)	4 mg/day (n = 59)	8 mg/day (n = 78)	12 mg/day (n = 46)	Total (n = 198)	
Mean age, years (SD)	29.7 (11.3)	30.3 (11.2)	29.5 (11.6)	27.5 (11.0)	29.2 (12.0)	28.7 (11.4)	28.9 (11.5)
Female, n (%)	43 (54.4)	10 (66.7)	26 (44.1)	37 (47.4)	22 (47.8)	95 (48.0)	130 (49.6)
Median BMI, kg/m^2^ (min, max)	21.3 (15.8, 34.1)	21.3 (17.5, 30.8)	22.8 (16.1, 31.3)	22.0 (16.0, 33.3)	22.1 (14.5, 31.7)	22.2 (14.5, 33.3)	22.1 (14.5, 34.1)
Seizure type, n (%)							
Focal aware without motor signs	8 (10.1)	5 (33.3)	5 (8.5)	9 (11.5)	3 (6.5)	22 (11.1)	25 (9.5)
Focal aware with motor signs	16 (20.3)	4 (26.7)	15 (25.4)	11 (14.1)	7 (15.2)	37 (18.7)	46 (17.6)
Focal impaired awareness	42 (53.2)	6 (40.0)	41 (69.5)	37 (47.4)	31 (67.4)	115 (58.1)	149 (56.9)
Focal with FBTCS	32 (40.5)	10 (66.7)	21 (35.6)	34 (43.6)	25 (54.3)	90 (45.5)	115 (43.9)
Tonic‐clonic	18 (22.8)	0 (0.0)	0 (0.0)	18 (23.1)	0 (0.0)	18 (9.1)	34 (13.0)
Myoclonic	2 (2.5)	0 (0.0)	0 (0.0)	5 (6.4)	0 (0.0)	5 (2.5)	7 (2.7)
Absence	4 (5.1)	0 (0.0)	0 (0.0)	2 (2.6)	0 (0.0)	2 (1.0)	6 (2.3)

Abbreviations: BMI, body mass index; FBTCS, focal to bilateral tonic‐clonic seizures; max, maximum; min, minimum; OLEx, open‐label extension; SD, standard deviation.

The full analysis set included 274 patients. Of these, 238 patients (placebo, n = 60; perampanel, n = 178) had FS, of which 120 patients had FBTCS during baseline (placebo, n = 31; perampanel, n = 89), and 36 patients (placebo, n = 18; perampanel, n = 18) had GTCS. Median (minimum, maximum) baseline seizure frequency per 28 days for placebo and perampanel 2, 4, 8, and 12 mg/day was as follows: 10.7 (3.1, 569.1), 15.3 (4.1, 284.2), 7.3 (3.1, 202.1), 7.7 (3.1, 80.8), and 8.1 (2.7, 295.3) for FS, respectively; and 3.7 (0.6, 20.7), 7.5 (0.7, 31.4), 4.3 (0.7, 24.7), 4.6 (0.7, 38.2), and 4.7 (0.7, 23.0) for FBTCS, respectively. For GTCS, median (minimum, maximum) baseline seizure frequency per 28 days was 2.4 (1.3, 9.3) for placebo and 3.0 (1.5, 18.5) for perampanel 8 mg/day.

### 3.2 Double‐blind studies: efficacy outcomes

**FIGURE 1:**
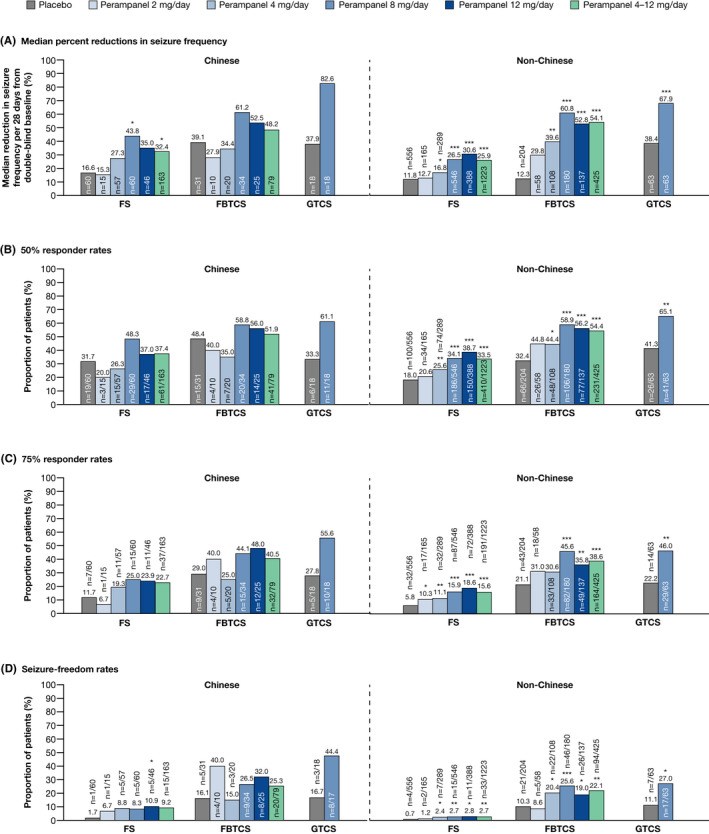
Double‐blind studies: (A) median percent reductions in seizure frequency per 28 days from baseline, (B) 50% responder rates, (C) 75% responder rates, and (D) seizure‐freedom rates for Chinese and non‐Chinese patients during maintenance (Full Analysis Set). *P < 0.05; **P < 0.01; ***P ≤ 0.0001 vs placebo. FBTCS, focal to bilateral tonic‐clonic seizures; FS, focal seizures; GTCS, generalized tonic‐clonic seizures

Median percent reductions in seizure frequency per 28 days for each seizure type are shown in Figure 1A. Across all seizure types, perampanel 8 mg/day was associated with numerically greater reductions in seizure frequency per 28 days compared with placebo in Chinese patients, although statistical significance vs placebo was only seen for FS. Perampanel 12 and 4‐12 mg/day also conferred numerically greater reductions in seizure frequency per 28 days vs placebo in Chinese patients with FS, with or without FBTCS. In non‐Chinese patients, all perampanel doses (except 2 mg/day) conferred significantly greater reductions in seizure frequency for FS, FBTCS, and GTCS. The smaller sample size for the Chinese cohort should be taken into consideration when interpreting these data. Median (95% CI) difference of perampanel vs placebo for each seizure type and cohort is provided in Table S2.

Responder and seizure‐freedom rates for Chinese and non‐Chinese patients are presented in Figure 1B‐D. Perampanel was associated with greater 50% and 75% responder rates, and seizure‐freedom rates for FS and FBTCS (at 8, 12, and 4‐12 mg/day) and for GTCS (at 8 mg/day) in Chinese and non‐Chinese patients, but most of these differences were not statistically significant vs placebo in the Chinese cohort.

### 3.3 Double‐blind studies: safety and tolerability outcomes

Treatment‐emergent adverse events were reported in 36 (45.6%) and 130 (65.7%) placebo‐ and perampanel‐treated Chinese patients (Table 2) compared with 434 (69.9%) and 1142 (78.7%) non‐Chinese patients, respectively (Table S3). Treatment‐related TEAEs were reported in 17 (21.5%) and 103 (52.0%) Chinese patients compared with 254 (40.9%) and 889 (61.2%) non‐Chinese patients, respectively. The most common TEAEs in perampanel‐treated Chinese patients were dizziness, upper respiratory tract infection, and increased weight (Table 2); the most common in non‐Chinese patients were dizziness, somnolence, and headache (Table S3). These TEAEs were all reported more frequently in patients receiving perampanel compared with those receiving placebo, with the exception of headache in non‐Chinese patients, which occurred at a similar rate between placebo‐ and perampanel‐treated patients. In Chinese patients, dizziness and increased weight were judged to be treatment‐related in 27.8% and 6.6% of patients (total perampanel), respectively (Table S4). The mean (standard deviation [SD]) percentage change from baseline in weight for Chinese patients with a TEAE of weight increased receiving any dose of perampanel was 7.1% (2.3) for patients aged <18 years (mean [SD] baseline weight: 56.9 [12.6] kg; mean [SD] end of treatment weight: 64.0 [12.8] kg) and 4.2% (2.7) for patients aged ≥18 years (mean [SD] baseline weight: 60.6 [12.8] kg; mean [SD] end of treatment weight: 64.9 [13.2] kg).

**TABLE 2:** Overview of TEAEs and most common TEAEs (occurring in ≥4% of patients in the total perampanel group) during the double‐blind and OLEx studies for Chinese patients (safety analysis set)

	Double‐blind studies						OLEx studies
	Placebo (n = 79)	Perampanel					Perampanel (n = 262)
		2 mg/day (n = 15)	4 mg/day (n = 59)	8 mg/day (n = 78)	12 mg/day (n = 46)	Total (n = 198)	
TEAEs, n (%)	36 (45.6)	11 (73.3)	32 (54.2)	50 (64.1)	37 (80.4)	130 (65.7)	213 (81.3)
Treatment‐related TEAEs, n (%)	17 (21.5)	8 (53.3)	22 (37.3)	41 (52.6)	32 (69.6)	103 (52.0)	189 (72.1)
Serious TEAEs, n (%)	3 (3.8)	0 (0.0)	2 (3.4)	2 (2.6)	3 (6.5)	7 (3.5)	20 (7.6)
TEAEs leading to study drug discontinuation, n (%)	3 (3.8)	0 (0.0)	1 (1.7)	3 (3.8)	5 (10.9)	9 (4.5)	30 (11.5)
Most common (≥4%) TEAEs, n (%)							
Dizziness	4 (5.1)	4 (26.7)	12 (20.3)	24 (30.8)	19 (41.3)	59 (29.8)	120 (45.8)
Upper respiratory tract infection	2 (2.5)	3 (20.0)	6 (10.2)	6 (7.7)	4 (8.7)	19 (9.6)	30 (11.5)
Weight increased	0 (0.0)	2 (13.3)	5 (8.5)	6 (7.7)	5 (10.9)	18 (9.1)	29 (11.1)
Nasopharyngitis	5 (6.3)	0 (0.0)	5 (8.5)	3 (3.8)	5 (10.9)	13 (6.6)	17 (6.5)
Somnolence	2 (2.5)	0 (0.0)	2 (3.4)	7 (9.0)	2 (4.3)	11 (5.6)	18 (6.9)
Irritability	0 (0.0)	2 (13.3)	2 (3.4)	7 (9.0)	2 (4.3)	13 (6.6)	15 (5.7)
Gait disturbance	0 (0.0)	0 (0.0)	2 (3.4)	2 (2.6)	3 (6.5)	7 (3.5)	16 (6.1)
Headache	5 (6.3)	0 (0.0)	3 (5.1)	3 (3.8)	1 (2.2)	7 (3.5)	11 (4.2)
Diarrhea	2 (2.5)	1 (6.7)	1 (1.7)	2 (2.6)	4 (8.7)	8 (4.0)	10 (3.8)
Fatigue	1 (1.3)	0 (0.0)	1 (1.7)	4 (5.1)	2 (4.3)	7 (3.5)	13 (5.0)
Protein urine present	2 (2.5)	0 (0.0)	0 (0.0)	2 (2.6)	1 (2.2)	3 (1.5)	11 (4.2)
Vision blurred	0 (0.0)	0 (0.0)	0 (0.0)	1 (1.3)	1 (2.2)	2 (1.0)	12 (4.6)
TEAEs related to hostility/aggression	2 (2.5)	2 (13.3)	2 (3.4)	10 (12.8)	8 (17.4)	22 (11.1)	–
Irritability	0 (0.0)	2 (13.3)	2 (3.4)	7 (9.0)	2 (4.3)	13 (6.6)	–
Aggression	0 (0.0)	0 (0.0)	0 (0.0)	2 (2.6)	2 (4.3)	4 (2.0)	–
Affect lability	0 (0.0)	0 (0.0)	0 (0.0)	1 (1.3)	1 (2.2)	2 (1.0)	–
Anger	0 (0.0)	0 (0.0)	0 (0.0)	0 (0.0)	2 (4.3)	2 (1.0)	–
Abnormal behavior	0 (0.0)	0 (0.0)	0 (0.0)	0 (0.0)	1 (2.2)	1 (0.5)	–
Personality change	0 (0.0)	0 (0.0)	0 (0.0)	0 (0.0)	1 (2.2)	1 (0.5)	–
Agitation	1 (1.3)	0 (0.0)	0 (0.0)	0 (0.0)	0 (0.0)	0 (0.0)	–
Personality disorder	1 (1.3)	0 (0.0)	0 (0.0)	0 (0.0)	0 (0.0)	0 (0.0)	–

Note

A TEAE is defined as an AE with an onset date, or a worsening in severity from baseline, on or after the first dose of study drug up to 30 days following study drug discontinuation. A patient with ≥2 AEs in the same system organ class or with the same preferred term is counted only once for that system organ class or preferred term.

Abbreviations: AE, adverse event; OLEx, open‐label extension; TEAE, treatment‐emergent adverse event.

The incidence of TEAEs related to hostility and/or aggression was low in Chinese patients, although more patients receiving perampanel 8 or 12 mg/day reported such events compared with placebo and lower perampanel doses. Irritability was reported more frequently in patients receiving perampanel compared with those receiving placebo (3.4%‐13.3% across perampanel doses vs 0% with placebo; Table 2).

TEAEs were more frequently reported with the higher perampanel doses (8 and 12 mg/day) compared with lower doses (2, 4, and 6 mg/day) in Chinese patients, with the exception of perampanel 10 mg/day, which showed a similar TEAE incidence to the lower doses (Table S5).

One Chinese patient in the placebo group from Study 335 died due to gastrointestinal hemorrhage and sudden cardiac death. Other serious TEAEs in Chinese patients were reported in 2 (2.5%) placebo‐treated patients (induced abortion and pneumonia) and 7 (3.5%) perampanel‐treated patients (anal abscess, FIAS, epilepsy, hemorrhoids, intervertebral disk protrusion, pneumonia, road traffic accident, and suicide attempt); the serious event of suicide attempt led to withdrawal. This is compared with 36 (5.8%) placebo‐treated and 81 (5.6%) perampanel‐treated non‐Chinese patients with serious TEAEs; 3 deaths occurred (1 patient receiving placebo and 2 receiving perampanel). Overall, 3 (3.8%) placebo‐treated and 9 (4.5%) perampanel‐treated Chinese patients reported TEAEs leading to discontinuation. These included convulsion, gastrointestinal hemorrhage (this was the same patient who died), and pregnancy in the placebo group, and abnormal behavior, aggression, dizziness, fear, hypersomnia, insomnia, irritability, personality change, suicide attempt (this was the same patient listed above who had a serious TEAE of suicide attempt), and abnormal thinking in the perampanel group. In the non‐Chinese population, TEAEs leading to discontinuation were reported in 29 (4.7%) placebo‐treated patients and 152 (10.5%) perampanel‐treated patients.

### 3.4 OLEx studies: patients

Overall, 262 Chinese patients entered the OLEx studies and were included in the safety analysis set. Patient demographics and baseline characteristics in the OLEx studies were generally similar to those in the double‐blind studies (Table 1). During baseline, 67 (25.6%), 124 (47.3%), and 71 (27.1%) patients were receiving one, two, and three concomitant ASMs, respectively; 152 (58.0%) patients were receiving EIASMs. The most common non‐EIASMs were valproic acid (n = 135 [51.5%]) and lamotrigine (n = 74 [28.2%]); the most common EIASM was carbamazepine (n = 93 [35.5%]).

The full analysis set included 260 patients; of these, 226 patients had FS, of which 118 had FBTCS during baseline, and 34 had GTCS. Median (minimum, maximum) preperampanel baseline seizure frequency per 28 days was 8.0 (1.6, 714.8) for FS, 4.2 (0.2, 38.2) for FBTCS, and 2.4 (0.5, 18.5) for GTCS.

### 3.5 OLEx studies: efficacy outcomes

**FIGURE 2:**
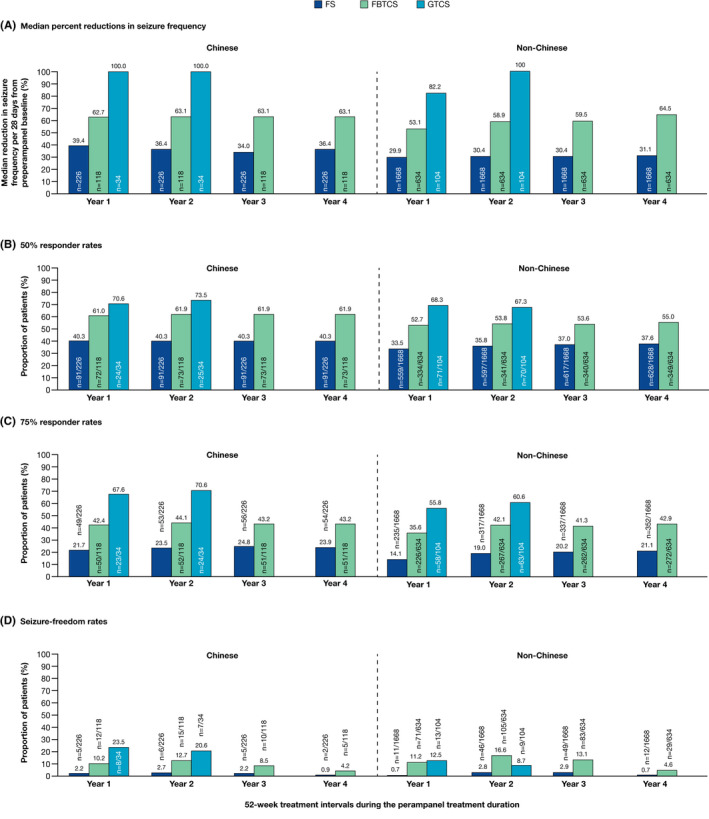
OLEx studies: (A) median percent reductions in seizure frequency per 28 days from preperampanel baseline, (B) 50% responder rates, (C) 75% responder rates, and (D) seizure‐freedom rates during the perampanel treatment duration by 52‐week treatment intervals for Chinese and non‐Chinese patients (full analysis set including early dropouts^a^). ^a^Last observation carried forward: patients who completed or withdrew from the study had their last year of treatment carried forward to later time points; for patients who were treated for <1 year, their entire treatment period was carried forward to later time points. FBTCS, focal to bilateral tonic‐clonic seizures; FS, focal seizures; GTCS, generalized tonic‐clonic seizures; OLEx, open‐label extension

Following long‐term adjunctive perampanel treatment, and including data for patients who dropped out of the study early, a reduction in the frequency of FS, FBTCS, and GTCS per 28 days was observed across the perampanel treatment duration in Chinese and non‐Chinese patients (Figure 2A). Responder (50% and 75%) and seizure‐freedom rates were maintained for up to 4 years for FS and FBTCS and up to 2 years for GTCS in both populations (Figure 2B‐D).

### 3.6 OLEx studies: safety and tolerability outcomes

Treatment‐emergent adverse events were reported in 213 (81.3%) Chinese patients vs 1636 (92.4%) non‐Chinese patients; 189 (72.1%) and 1440 (81.3%) patients had treatment‐related TEAEs, respectively (Table 2, Table S3). The most common TEAEs were dizziness, upper respiratory tract infection, and weight increased (Chinese patients), and dizziness, somnolence, and headache (non‐Chinese patients). There were no deaths of Chinese patients and 15 deaths of non‐Chinese patients. Serious TEAEs were reported in 20 (7.6%) Chinese patients and 355 (20.0%) non‐Chinese patients. In Chinese patients, these included anal abscess, ankle fracture, appendicitis, central nervous system lesion, cerebral infarction, FIAS, craniocerebral injury, dizziness, epilepsy, fibula fracture, induced abortion, intentional overdose, intervertebral disk protrusion, gastritis, hemorrhoids, lumbar vertebral fracture, mental disorder, pneumonia, pneumothorax, pregnancy, psychiatric symptom, road traffic accident, suicide attempt, and status epilepticus. Thirty (11.5%) Chinese patients experienced TEAEs leading to discontinuation vs 326 (18.4%) non‐Chinese patients. In Chinese patients, these included abnormal behavior, abnormal thinking, aggression, anger, blurred vision, cerebral infarction, dizziness, fatigue, fear, gait disturbance, head injury, hepatic function abnormal, hypersomnia, increased appetite, increased gamma‐glutamyltransferase, induced abortion, insomnia, irritability, mania, mood swings, nasal congestion, personality change, pregnancy, suicide attempt, vertigo, and weight increased.

## 4 DISCUSSION

In this post hoc analysis, once‐daily adjunctive perampanel was efficacious and well tolerated in patients from China with FS, with or without FBTCS, or GTCS. Seizure control established during the double‐blind studies was maintained for up to 4 years for FS and FBTCS and up to 2 years for GTCS during OLEx studies.

Previous international studies of adjunctive perampanel have predominantly included Caucasian populations.^18^, ^19^, ^20^, ^22^ To identify regional differences between responses to perampanel, a pooled analysis for FS (with or without FBTCS) using data from Studies 304, 305, 306, and 335 assessed the efficacy and safety of adjunctive perampanel in Asian vs non‐Asian populations.^25^ Adjunctive perampanel 8 and 12 mg/day were consistently associated with significantly greater median percent reductions in seizure frequency (Asian: both *P* < 0.0001; non‐Asian: *P* < 0.0001 and *P < *0.001, respectively) and 50% responder rates (Asian: both *P* < 0.0001; non‐Asian: *P* < 0.0001 and *P < *0.001, respectively) compared with placebo in both populations,^25^ and there were no significant differences in efficacy between Asian and non‐Asian populations.^25^

Since licensing of new ASMs in Asia is completed at the individual country level,^2^ our post hoc analysis assessed outcomes in patients from China to determine if responses to adjunctive perampanel are consistent with those at a regional and international level. Our results for FS and FBTCS showed adjunctive perampanel 8, 12, and 4‐12 mg/day were associated with greater reductions in seizure frequency compared with placebo, as well as greater 50% responder and seizure‐freedom rates. In addition, perampanel 8 mg/day was also shown to confer additional efficacy for GTCS compared with placebo. Similar patterns of response to perampanel and placebo were generally observed in Chinese and non‐Chinese. However, in the Chinese cohort differences between perampanel and placebo were often not significant unlike in the non‐Chinese cohort, which may be attributable to the smaller sample size in the Chinese cohort. Despite this, our results are generally consistent with those previously reported in Asian and non‐Asian populations with FS or GTCS,^18^, ^19^, ^20^, ^21^, ^22^, ^25^ suggesting Chinese race does not differ in regard to perampanel efficacy. Furthermore, previous pharmacokinetic/pharmacodynamic analyses have shown that Chinese race does not affect the relationship between perampanel exposure and clinical response in patients with FS,^26^ and results of our post hoc analysis provide further evidence supporting this.

Treatment‐emergent adverse events were reported in 45.6% of placebo‐treated and 65.7% of perampanel (2‐12 mg/day)‐treated Chinese patients across double‐blind studies, which was slightly lower than rates in non‐Chinese patients (69.9% and 78.7%, respectively). TEAE incidence in patients from China was also similar, albeit slightly lower, than previously reported in patients with FS from Study 335 (placebo, 66.5%; perampanel 4‐12 mg/day, 76.5%),^21^ a pooled analysis of Studies 304, 305, and 306 (placebo, 66.5%; perampanel 2‐12 mg/day, 77.0%),^27^ and in patients with GTCS from Study 332 (placebo, 72.0%; perampanel 8 mg/day, 82.7%).^22^ The most common TEAE reported with perampanel was dizziness in both Chinese and non‐Chinese patients, which is consistent with previous perampanel studies.^21^, ^22^, ^27^ Incidences of serious TEAEs and TEAEs leading to discontinuation were low in Chinese patients and similar between treatment groups (placebo and total perampanel: all <5%); rates were also lower than in non‐Chinese patients. These results demonstrate that the safety and tolerability profile of perampanel in Chinese patients are consistent with non‐Chinese patients and the known safety profile of perampanel.^7^, ^8^

Since patients retained on treatment at later time points are likely to include those with favorable tolerability and efficacy responses, dropout analyses were conducted in the current analysis to account for potential selection bias at later treatment intervals during OLEx studies. Improvements in seizure control were observed following long‐term perampanel treatment; however, improvements were particularly notable for FBTCS and GTCS. Consistent with our results, perampanel was shown to be particularly effective against FBTCS in Study 307.^24^ The additional efficacy of perampanel against generalized seizure types may be related to its mechanism of action as a selective AMPA receptor antagonist.^28^ AMPA receptors have been implicated in several disorders characterized by overexcitation^29^, ^30^ and there is increasing evidence suggesting generalized seizures are characterized by abnormalities in cortical hyperexcitability that are affected by ASM use.^31^, ^32^

The long‐term safety profile of adjunctive perampanel in patients from China was consistent with that in non‐Chinese patients and with the known safety profile of perampanel. No new safety signals emerged during long‐term treatment. These data support the recent approval of perampanel as adjunctive treatment for FS in patients with epilepsy aged ≥12 years and provide evidence suggesting perampanel may also be a suitable treatment option for Chinese patients with GTCS.

Potential limitations of this analysis include those inherent to post hoc analyses, as well as small patient numbers in some treatment groups meaning that statistical analysis may not be robust. The open‐label nature of OLEx studies means no placebo data are available with which to compare outcomes during long‐term treatment.

## 5 CONCLUSION

This post hoc analysis provides the first perampanel data in a population of Chinese patients with epilepsy. Our results demonstrate that the short‐ and long‐term efficacy, safety, and tolerability profile of adjunctive perampanel in Chinese patients with FS, with or without FBTCS, or GTCS are consistent with non‐Chinese patients and those reported during global phase III studies. These findings provide further guidance for the use of perampanel in Chinese patients with epilepsy in real‐life settings and the personalization of treatment decisions for this population.

## DISCLOSURE

Liao Weiping, Zhou Dong, and Hong Zhen do not have any real or apparent conflicts of interest to disclose in relation to this work. Anna Patten is an employee of Eisai Ltd. Amitabh Dash is an employee of Eisai Singapore Pte. Ltd. Manoj Malhotra is an employee of Eisai Inc.

## AUTHOR CONTRIBUTIONS

All authors provided substantial contributions to the conception and/or design of the post hoc analysis, the acquisition of data, or data analysis. All authors were involved in the interpretation of the results, the drafting, reviewing, and approval of the manuscript and in the decision to submit the article for publication. All authors also confirm accountability for the accuracy and integrity of the work.

## Supporting information

Table S1‐S5

## Data Availability

The data that support the findings of this study are available from the corresponding author upon reasonable request.
