# Supplementary material for: Efficacy, safety, and tolerability of adjunctive perampanel in patients from China with focal seizures or generalized tonic‐clonic seizures: Post hoc analysis of phase III double‐blind and open‐label extension studies
Source: CNS Neurosci Ther. 2020 Sep 8;27(3):330–40. doi: 10.1111/cns.13458 (PMC7871786; doi:10.1111/cns.13458)
Supplement: Supplementary file 1 — Table S1‐S5 [file CNS-27-330-s001.pdf]

SUPPLEMENTARY MATERIAL

SUPPLEMENTARY TABLE 1 Overview of double-blind and OLEx studies

Study number	Study design	Study periods	Included centers in China?
**Double-blind studies**			
Study 304 (NCT00699972)	International, Phase III, multicenter, randomized (1:1:1), double-blind, placebo-controlled study to assess efficacy and safety of adjunctive perampanel 8 or 12 mg/day in patients (aged ≥12 years) with drug-resistant FS, with or without FBTCS, despite treatment with 1–3 other ASMs	• 6-week baseline period<br>• 19-week double-blind treatment phase (6-week titration; 13-week maintenance)<br>• 4-week follow-up period	No
Study 305 (NCT00699582)	International, Phase III, multicenter, randomized (1:1:1), double-blind, placebo-controlled study to assess efficacy and safety of adjunctive perampanel 8 or 12 mg/day in patients (aged ≥12 years) with drug-resistant FS, with or without FBTCS, despite treatment with 1–3 other ASMs	• 6-week baseline period<br>• 19-week double-blind treatment phase (6-week titration; 13-week maintenance)<br>• 4-week follow-up period	No
Study 306 (NCT00700310)	International, Phase III, multicenter, randomized (1:1:1:1), double-blind, placebo-controlled study to assess efficacy and safety of adjunctive perampanel 2, 4, or 8 mg/day in patients (aged ≥12 years) with drug-resistant FS, with or without FBTCS, despite treatment with 1–3 other ASMs	• 6-week baseline period<br>• 19-week double-blind treatment phase (6-week titration; 13-week maintenance)<br>• 4-week follow-up period	Yes
Study 335 (NCT01618695)	Asia-Pacific, Phase III, multicenter, randomized (1:1:1:1), double-blind, placebo-controlled, parallel-group study to assess efficacy, safety, and tolerability of adjunctive perampanel 4, 8, or 12 mg/day in	• 6-week baseline period	Yes

	patients (aged ≥12 years) with drug-resistant FS, with or without FBTCS, despite treatment with 1–3 other ASMs	• 19-week double-blind treatment phase (6-week titration; 13-week maintenance)<br>• 4-week follow-up period	
Study 332 (NCT01393743)	International, Phase III multicenter, randomized (1:1), double-blind, placebo-controlled, parallel-group study to assess efficacy and safety of adjunctive perampanel 8 mg/day (target daily dose) in patients (aged ≥12 years) with drug-resistant GTCS in IGE, despite treatment with 1–3 other ASMs	• Pre-randomization phase, including screening (≤4 weeks) and baseline (4 or 8 weeks)<br>• 17-week double-blind treatment phase (4-week titration; 13-week maintenance)<br>• 4-week follow-up period	Yes
**OLEx studies**			
OLEx Study 307 (NCT00735397)	Open-label, international, extension study: patients aged ≥12 years with FS with or without FBTCS who completed one of the following double-blind Phase III studies were eligible for enrolment: Study 304, Study 305, and Study 306	• Blinded 16-week conversion period<br>○ Patients who previously received placebo during the double-blind studies converted in a blinded manner to adjunctive perampanel. Perampanel dose up-titrated in 2-mg increments biweekly from the dose on which they completed the double-blind study (or from 2 mg for patients previously receiving placebo), to a maximum of 12 mg/day, based on tolerability and seizure control<br>• 256-week open-label maintenance period<br>○ Patients unblinded to study treatment and remained on the optimal perampanel dose established during the conversion period<br>○ Adjustment of perampanel dose during the maintenance period permitted at the investigator's discretion, based on efficacy and tolerability	Yes

Study 335 OLEx (NCT01618695)	Open-label, international, extension study of Study 335 in patients aged ≥12 years with drug-resistant FS, with or without FBTCS, despite treatment with 1–3 other ASMs	• Blinded 6-week conversion period<br>○ Patients who previously received placebo during the double-blind studies converted in a blinded manner to adjunctive perampanel. Perampanel dose up-titrated in 2-mg increments weekly from the dose on which they completed the double-blind study (or from 2 mg for patients previously receiving placebo), to a maximum of 12 mg/day, based on tolerability and seizure control<br>• ≥46 week open-label maintenance period<br>○ Patients unblinded to study treatment and remained on the optimal perampanel dose established during the conversion period<br>○ Adjustment of perampanel dose during the maintenance period permitted at the investigator's discretion, based on efficacy and tolerability	Yes
Study 332 OLEx (NCT01393743)	Open-label, international, extension study of Study 332 in patients aged ≥12 years with drug-resistant GTCS in IGE, despite treatment with 1–3 other ASMs	• Blinded 6-week conversion period<br>○ Patients who previously received placebo during the double-blind studies were converted in a blinded manner to adjunctive perampanel. Perampanel dose was up-titrated in 2-mg increments weekly from the dose on which they completed the double-blind study (or from 2 mg for patients previously receiving placebo), to a maximum of 12 mg/day, based on tolerability and seizure control<br>• 136-week open-label maintenance period<br>○ Patients unblinded to study treatment and remained on the optimal perampanel dose established during the conversion period	Yes

		○ Adjustment of perampanel dose during the maintenance period permitted at the investigator's discretion, based on efficacy and tolerability

Abbreviations: ASM, anti-seizure medication; FBTCS, focal to bilateral tonic-clonic seizures; FS, focal seizures; GTCS, generalized tonic-clonic seizures; IGE, idiopathic generalized epilepsy; OLEx, open-label extension.

SUPPLEMENTARY TABLE 2 Double-blind studies: median (95% CI) difference between placebo and perampanel for percent reductions in seizure frequency per 28 days from baseline for Chinese and non-Chinese patients (Full Analysis Set)

**Median (95% CI) difference from placebo**	**Perampanel**				
	**2 mg/day**	**4 mg/day**	**8 mg/day**	**12 mg/day**	**4–12 mg/day**
**Chinese patients**					
FS	5.4 (-27.0, 37.0)	-8.4 (-26.4, 10.4)	-21.9 (-40.7, -2.7)	-18.4 (-38.6, 1.8)	-15.8, (-31.3, -0.5)
FBTCS	0.0 (-55.6, 49.4)	0.4 (-33.9, 25.6)	-18.0 (-48.1, 7.5)	-19.2 (-51.6, 0.7)	-12.4 (-40.9, 6.1)
GTCS	–	–	-28.2 (-63.7, 7.5)	–	–
**Non-Chinese patients**					
FS	-3.4 (-11.2, 4.3)	-7.9 (-14.2, -1.7)	-17.0 (-22.3, 11.9)	-19.8 (-25.5, -14.0)	-15.8 (-20.2, -11.3)
FBTCS	-15.2 (-34.9, 3.2)	-21.6 (-36.6, -6.7)	-37.2 (-50.5, -25.5)	-33.3 (-47.7, -20.6)	-31.6 (-42.6, -22.1)
GTCS	–	–	-30.8 (-47.1, -14.3)	–	–

Abbreviations: CI, confidence interval; FBTCS, focal to bilateral tonic-clonic seizures; FS, focal seizures; GTCS, generalized tonic-clonic seizures.

SUPPLEMENTARY TABLE 3 Overview of TEAEs and most common TEAEs (occurring in ≥4% of patients in the total perampanel group) during the double-blind and OLEx studies for non-Chinese patients (Safety Analysis Set)

	**Double-blind studies**						**OLEx studies**
	**Placebo (n=621)**	**Perampanel**					**Perampanel (n=1771)**
		**2 mg/day (n=165)**	**4 mg/day (n=289)**	**8 mg/day (n=609)**	**12 mg/day (n=389)**	**Total (n=1452)**	
**TEAEs, n (%)**	434 (69.9)	100 (60.6)	200 (69.2)	496 (81.4)	346 (88.9)	1142 (78.7)	1636 (92.4)
**Treatment-related TEAEs, n (%)**	254 (40.9)	59 (35.8)	136 (47.1)	396 (65.0)	298 (76.6)	889 (61.2)	1440 (81.3)
**Serious TEAEs, n (%)**	36 (5.8)	6 (3.6)	10 (3.5)	35 (5.7)	30 (7.7)	81 (5.6)	355 (20.0)
**TEAEs leading to study drug discontinuation, n (%)**	29 (4.7)	12 (7.3)	12 (4.2)	59 (9.7)	69 (17.7)	152 (10.5)	326 (18.4)
**Most common (≥4%) TEAEs, n (%)**							
Dizziness	51 (8.2)	14 (8.5)	56 (19.4)	189 (31.0)	166 (42.7)	425 (29.3)	778 (43.9)
Somnolence	56 (9.0)	22 (13.3)	42 (14.5)	100 (16.4)	75 (19.3)	239 (16.5)	405 (22.9)
Headache	66 (10.6)	16 (9.7)	28 (9.7)	69 (11.3)	43 (11.1)	156 (10.7)	306 (17.3)
Nasopharyngitis	46 (7.4)	7 (4.2)	27 (9.3)	51 (8.4)	29 (7.5)	114 (7.9)	266 (15.0)

Fatigue	30 (4.8)	8 (4.8)	16 (5.5)	50 (8.2)	38 (9.8)	112 (7.7)	203 (11.5)
Irritability	16 (2.6)	5 (3.0)	13 (4.5)	41 (6.7)	37 (9.5)	96 (6.6)	106 (6.0)
Nausea	29 (4.7)	4 (2.4)	8 (2.8)	34 (5.6)	30 (7.7)	76 (5.2)	144 (8.1)
Upper respiratory tract infection	23 (3.7)	8 (4.8)	8 (2.8)	26 (4.3)	17 (4.4)	59 (4.1)	135 (7.6)
Weight increased	10 (1.6)	1 (0.6)	9 (3.1)	24 (3.9)	12 (3.1)	46 (3.2)	171 (9.7)
Fall	18 (2.9)	2 (1.2)	3 (1.0)	25 (4.1)	26 (6.7)	56 (3.9)	127 (7.2)
Convulsion	19 (3.1)	3 (1.8)	6 (2.1)	16 (2.6)	11 (2.8)	36 (2.5)	123 (6.9)
Vomiting	21 (3.4)	3 (1.8)	4 (1.4)	22 (3.6)	14 (3.6)	43 (3.0)	106 (6.0)
Insomnia	23 (3.7)	2 (1.2)	4 (1.4)	20 (3.3)	13 (3.3)	39 (2.7)	105 (5.9)
Vertigo	6 (1.0)	6 (3.6)	10 (3.5)	21 (3.4)	14 (3.6)	51 (3.5)	94 (5.3)
Diarrhea	26 (4.2)	1 (0.6)	5 (1.7)	20 (3.3)	12 (3.1)	38 (2.6)	99 (5.6)
Ataxia	1 (0.2)	0 (0.0)	3 (1.0)	16 (2.6)	27 (6.9)	46 (3.2)	106 (6.0)
Contusion	14 (2.3)	1 (0.6)	4 (1.4)	19 (3.1)	11 (2.8)	35 (2.4)	97 (5.5)
Gait disturbance	10 (1.6)	1 (0.6)	2 (0.7)	20 (3.3)	16 (4.1)	39 (2.7)	91 (5.1)
Anxiety	7 (1.1)	4 (2.4)	4 (1.4)	21 (3.4)	10 (2.6)	39 (2.7)	94 (5.3)
Balance disorder	3 (0.5)	0 (0.0)	4 (1.4)	26 (4.3)	12 (3.1)	42 (2.9)	90 (5.1)
Pyrexia	9 (1.4)	6 (3.6)	4 (1.4)	16 (2.6)	6 (1.5)	32 (2.2)	103 (5.8)

Back pain	10 (1.6)	1 (0.6)	4 (1.4)	10 (1.6)	14 (3.6)	29 (2.0)	100 (5.6)
Influenza	16 (2.6)	2 (1.2)	6 (2.1)	13 (2.1)	7 (1.8)	28 (1.9)	95 (5.4)
Rash	9 (1.4)	2 (1.2)	6 (2.1)	16 (2.6)	13 (3.3)	37 (2.5)	81 (4.6)
Dysarthria	1 (0.2)	0 (0.0)	2 (0.7)	17 (2.8)	13 (3.3)	32 (2.2)	82 (4.6)
Aggression	2 (0.3)	1 (0.6)	5 (1.7)	11 (1.8)	12 (3.1)	29 (2.0)	85 (4.8)
Depression	9 (1.4)	0 (0.0)	1 (0.3)	7 (1.1)	8 (2.1)	16 (1.1)	95 (5.4)
Diplopia	4 (0.6)	1 (0.6)	2 (0.7)	8 (1.3)	9 (2.3)	20 (1.4)	72 (4.1)
Laceration	0 (0.0)	0 (0.0)	0 (0.0)	2 (0.3)	1 (0.3)	3 (0.2)	70 (4.0)

Note: A TEAE is defined as an AE with an onset date, or a worsening in severity from baseline, on or after the first dose of study drug up to 30 days following study drug discontinuation. A patient with ≥2 AEs in the same system organ class or with the same preferred term is counted only once for that system organ class or preferred term.

Abbreviations: AE, adverse event; OLEx, open-label extension; TEAE, treatment-emergent adverse event.

SUPPLEMENTARY TABLE 4 Overview of treatment-related TEAEs (occurring in ≥2% of Chinese patients in the total perampanel group) during the double-blind studies (Safety Analysis Set)

	**Double-blind studies**					
	**Placebo (n=79)**	**Perampanel**				
		**2 mg/day (n=15)**	**4 mg/day (n=59)**	**8 mg/day (n=78)**	**12 mg/day (n=46)**	**Total (n=198)**
**Any treatment-related TEAE, n (%)**	17 (21.5)	8 (53.3)	22 (37.3)	41 (52.6)	32 (69.6)	103 (52.0)
**Most common (≥2%) treatment-related TEAEs, n (%)**						
Dizziness	3 (3.8)	3 (20.0)	11 (18.6)	23 (29.5)	18 (39.1)	55 (27.8)
Weight increased	0 (0.0)	1 (6.7)	4 (6.8)	4 (5.1)	4 (8.7)	13 (6.6)
Irritability	0 (0.0)	2 (13.3)	2 (3.4)	5 (6.4)	2 (4.3)	11 (5.6)
Somnolence	1 (1.3)	0 (0.0)	2 (3.4)	5 (6.4)	1 (2.2)	8 (4.0)
Vertigo	0 (0.0)	0 (0.0)	0 (0.0)	5 (6.4)	2 (4.3)	7 (3.5)
Gait disturbance	0 (0.0)	0 (0.0)	1 (1.7)	2 (2.6)	3 (6.5)	6 (3.0)
Fatigue	0 (0.0)	0 (0.0)	1 (1.7)	2 (2.6)	2 (4.3)	5 (2.5)
Aggression	0 (0.0)	0 (0.0)	0 (0.0)	2 (2.6)	2 (4.3)	4 (2.0)
Headache	1 (1.3)	0 (0.0)	0 (0.0)	3 (3.8)	1 (2.2)	4 (2.0)

Note: A TEAE is defined as an AE with an onset date, or a worsening in severity from baseline, on or after the first dose of study drug up to 30 days following study drug discontinuation. A patient with ≥2 AEs in the same system organ class or with the same preferred term is counted only once for that system organ class or preferred term.

Abbreviations: AE, adverse event; TEAE, treatment-emergent adverse event.

SUPPLEMENTARY TABLE 5 Overview of TEAEs and most common TEAEs (occurring in ≥4% of patients in the total perampanel group) during the double-blind and OLEx studies for Chinese patients by dose at time of onset (Safety Analysis Set)

	**Double-blind studies**						
	**Placebo (n=79)**	**Perampanel**					
		**2 mg/day (n=198)**	**4 mg/day (n=183)**	**6 mg/day (n=124)**	**8 mg/day (n=124)**	**10 mg/day (n=45)**	**12 mg/day (n=43)**
**TEAEs, n (%)**	36 (45.6)	44 (22.2)	51 (27.9)	14 (11.3)	59 (47.6)	9 (20.0)	24 (55.8)
**Most common (≥4%) TEAEs, n (%)**							
Dizziness	4 (5.1)	17 (8.6)	15 (8.2)	6 (4.8)	22 (17.7)	4 (8.9)	6 (14.0)
Upper respiratory tract infection	2 (2.5)	5 (2.5)	8 (4.4)	0 (0.0)	6 (4.8)	0 (0.0)	3 (7.0)
Nasopharyngitis	5 (6.3)	2 (1.0)	5 (2.7)	1 (0.8)	3 (2.4)	1 (2.2)	2 (4.7)
Weight increased	0 (0.0)	7 (3.5)	5 (2.7)	0 (0.0)	5 (4.0)	0 (0.0)	1 (2.3)
Somnolence	2 (2.5)	3 (1.5)	3 (1.6)	1 (0.8)	6 (4.8)	1 (2.2)	0 (0.0)
Irritability	0 (0.0)	5 (2.5)	2 (1.1)	0 (0.0)	6 (4.8)	0 (0.0)	1 (2.3)
Headache	5 (6.3)	2 (1.0)	3 (1.6)	0 (0.0)	1 (0.8)	0 (0.0)	1 (2.3)
Gait disturbance	0 (0.0)	1 (0.5)	1 (0.5)	2 (1.6)	2 (1.6)	0 (0.0)	2 (4.7)
Insomnia	4 (5.1)	1 (0.5)	1 (0.5)	0 (0.0)	1 (0.8)	0 (0.0)	0 (0.0)

Hepatic function abnormal	1 (1.3)	1 (0.5)	1 (0.5)	0 (0.0)	0 (0.0)	0 (0.0)	2 (4.7)
Aggression	0 (0.0)	0 (0.0)	0 (0.0)	0 (0.0)	2 (1.6)	0 (0.0)	2 (4.7)
Electrocardiogram Qt prolonged	0 (0.0)	0 (0.0)	1 (0.5)	0 (0.0)	0 (0.0)	0 (0.0)	2 (4.7)

Note: A TEAE is defined as an AE with an onset date, or a worsening in severity from baseline, on or after the first dose of study drug up to 30 days following study drug discontinuation. A patient with ≥2 AEs in the same system organ class or with the same preferred term is counted only once for that system organ class or preferred term. A patient that has the same event at different doses would appear under each dose that the event occurs.

Abbreviations: AE, adverse event; OLEx, open-label extension; TEAE, treatment-emergent adverse event.
